# Rapid detection of temocillin resistance in Enterobacterales

**DOI:** 10.1093/jac/dkad243

**Published:** 2023-08-07

**Authors:** Jacqueline Findlay, Laurent Poirel, Patrice Nordmann

**Affiliations:** Medical and Molecular Microbiology, Faculty of Science and Medicine, University of Fribourg, Chemin du Musée 18, CH-1700 Fribourg, Switzerland; Medical and Molecular Microbiology, Faculty of Science and Medicine, University of Fribourg, Chemin du Musée 18, CH-1700 Fribourg, Switzerland; Swiss National Reference Center for Emerging Antibiotic Resistance (NARA), University of Fribourg, Fribourg, Switzerland; INSERM European Unit (IAME, France), University of Fribourg, Fribourg, Switzerland; Medical and Molecular Microbiology, Faculty of Science and Medicine, University of Fribourg, Chemin du Musée 18, CH-1700 Fribourg, Switzerland; Swiss National Reference Center for Emerging Antibiotic Resistance (NARA), University of Fribourg, Fribourg, Switzerland; INSERM European Unit (IAME, France), University of Fribourg, Fribourg, Switzerland; Institute for Microbiology, University of Lausanne and University Hospital Centre, Lausanne, Switzerland

Temocillin is a semi-synthetic 6-α-methoxy derivative of ticarcillin, first developed in 1981.^1^ Despite being developed over 40 years ago, temocillin’s use as an antimicrobial agent was largely overlooked as a treatment option for infections caused by Gram-negative bacteria due to its poor activity against non-fermenters, including *Pseudomonas* spp. and *Acinetobacter* spp.^1^ Temocillin has been demonstrated to have an affinity for penicillin-binding protein 3 in *Escherichia coli*^2^ and remarkable stability against a plethora of β-lactamases including ESBLs and AmpCs (both plasmid and chromosomal);^3,4^ however, it is not clinically useful against bacteria producing class B (e.g. NDM) or class D (e.g. OXA-48-type) carbapenemases, because those latter enzymes readily hydrolyse this antibiotic, often leading to high MICs of temocillin.^5^ A recent study evaluating the use of temocillin against ESBL- and AmpC-producing Enterobacterales confirmed the excellent activity of temocillin, and the few high MICs observed were linked mostly to the carriage of multiple β-lactamases by corresponding isolates rather than any single β-lactamase type.^6^ In recent years, a number of countries in Europe, including Belgium, France, Luxembourg and the UK, have revived the use of this antibiotic, predominantly for the treatment of urinary tract infections but also for bloodstream and lower respiratory tract infections.^7,8^ Subsequently, the surveillance of resistance to temocillin is essential and can be easily determined using either routine broth microdilution or disc diffusion testing; however, such tests require 18–24 h to achieve a result. In this study, we designed and evaluated a rapid (∼4 h) test that can determine resistance or susceptibility to temocillin in Enterobacterales.

A total of 100 clinical Enterobacterales isolates were tested, comprising 55 *E. coli*, 33 *Klebsiella pneumoniae*, 6 *Enterobacter cloacae*, 4 *Klebsiella aerogenes* and 2 *Klebsiella oxytoca* isolates (Table S1, available as Supplementary data at *JAC* Online). Isolates were submitted to the Swiss National Reference Center for Emerging Antibiotic Resistance (NARA) from hospitals and clinics throughout Switzerland, and speciated by either MALDI-TOF MS (Bruker Microflex LT, Bruker Daltonik GmbH, Bremen, Germany) or API-20E tests (bioMérieux, https://www.biomerieux.com). Susceptibility testing was performed by broth microdilution according to CLSI guidelines,^9^ and resistance was determined according to EUCAST breakpoints (R > 16 mg/L),^10^ because there are no available guidelines for temocillin resistance according to CLSI. This identified that 38 tested isolates were temocillin susceptible, with MICs ranging from 2 to 16 mg/L, and the remaining 62 were resistant, with MICs ranging from 32 to >256 mg/L. The β-lactamase content of the tested isolates was determined by multiplex PCRs targeting CTX-M, CMY, DHA, SHV, TEM and OXA-1-like encoding genes,^11^ and the carbapenemase gene content had previously been determined by PCR, and subsequent Sanger sequencing of amplicons by NARA.^12^

The Rapid Temocillin NP test solution was prepared by mixing 6.25 g of cation-adjusted Mueller–Hinton (MH) broth (CA-MHB) powder (Bio-Rad), 0.0125 g of phenol red (Sigma Aldrich) and 225 mL of distilled water. The pH was adjusted to 7.3 by adding drops of 1 mol/L hydrochloric acid prior to autoclaving. After cooling to room temperature, 25 mL of a 10% anhydrous D(+)-glucose (Roth, Karlsruhe, Germany) (sterilized by filtration) was added, resulting in the final concentrations of 2.5% CA-MHB, 0.005% phenol red indicator and 1% glucose. The temocillin test solution was prepared with 21.3 mg/L of temocillin and 150 µL was added into one well of a 96-well polystyrene plate; 150 µL of test solution without temocillin was added into a second well. Inoculums were prepared from overnight cultures, grown on URISelect 4 agar (Bio-Rad, https://www.bio-rad.com), as follows: a 0.5 MacFarland suspension was prepared then diluted 1/10 in fresh MH broth; 50 µL of this suspension was then used to inoculate the plates (approx. 1.5 × 10^7^ bacterial cells per well), resulting in a final temocillin concentration of 16 mg/L, before incubation at 37°C for 4 h. Negative control wells, both with and without temocillin, were inoculated with 50 µL 0.85% NaCl, diluted 1/10 in fresh MH broth, instead of the bacterial suspension. After 3 h and 4 h, plates were read and a colour change from red to yellow was interpreted as positive (resistant), confirming acid production via the metabolism of glucose in the test solution, whereas red was interpreted as negative (susceptible) (Figure 1).

**Figure 1.**
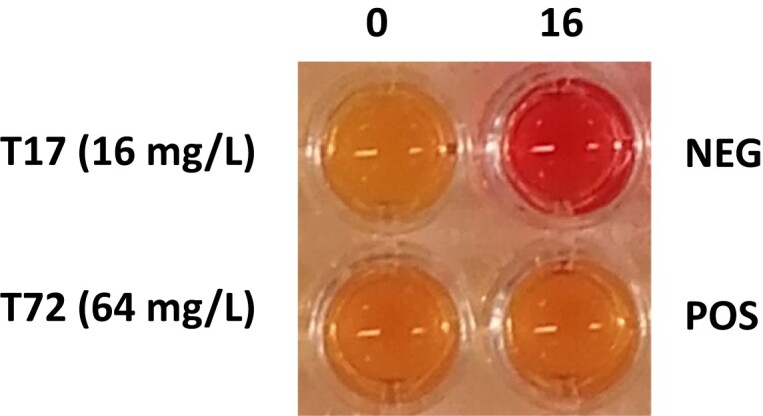
An example of a negative and positive test result. 0 = test solution containing no temocillin; 16 = test solution containing temocillin at a final concentration of 16 mg/L. T17, *E. coli* isolate with an MIC of 16 mg/L, T72, *K. pneumoniae* isolate with an MIC of 64 mg/L. This figure appears in colour in the online version of *JAC* and in black and white in the print version of *JAC*.

The test presented a single false-negative result, this being an *E. coli* isolate (T32) harbouring *bla*_TEM_ as a sole acquired ß-lactamase gene, exhibiting an MIC of temocillin at 32 mg/L, therefore corresponding to a 100% specificity and 98.4% sensitivity. Most of the isolates exhibiting high-level temocillin resistance (MIC ≥64 mg/L) resulted in a positive test result after only 3 h; however, those with resistant MICs closer to the breakpoint (32 mg/L) required 4 h. The test was shown to be effective regardless of the Enterobacterales species, β-lactamase content and overall mechanism of resistance. This rapid test could be easily implemented in a clinical laboratory and can be set up alongside routine antimicrobial susceptibility testing methodologies (AST), but providing a result 14–20 h earlier than traditional AST and potentially sparing the use of other β-lactams such as the carbapenems. These results show that as the use of temocillin to treat Gram-negative infections becomes more commonplace, such a test can prove useful in determining targeted rather than simply empirical therapy in a relatively short time frame.

## Supplementary Material

dkad243_Supplementary_Data

## Supplementary data

Table S1 is available as Supplementary data at *JAC* Online.
