# Supplementary material for: Rapid detection of temocillin resistance in Enterobacterales
Source: J Antimicrob Chemother. 2023 Aug 7;78(11):2770–1. doi: 10.1093/jac/dkad243 (PMC10631819; doi:10.1093/jac/dkad243)
Supplement: dkad243_Supplementary_Data [file dkad243_supplementary_data.docx]

| **Isolate** | **Species** | **Beta-lactamases** | **Temocillin MIC (mg/L)** | **Test Result** | **Match?** |
| --- | --- | --- | --- | --- | --- |
| T1 | *Escherichia coli* | CTX-M G1 | 2 | S | Y |
| T2 | *Escherichia coli* | CTX-M G2 | 2 | S | Y |
| T3 | *Escherichia coli* | CTX-M G1, OXA | 8 | S | Y |
| T4 | *Escherichia coli* | CTX-M G1, TEM | 8 | S | Y |
| T5 | *Escherichia coli* | CTX-M G1, TEM | 8 | S | Y |
| T6 | *Escherichia coli* | CTX-M G9, TEM | 8 | S | Y |
| T7 | *Escherichia coli* | OXA | 8 | S | Y |
| T8 | *Escherichia coli* | TEM | 8 | S | Y |
| T9 | *Escherichia coli* |  | 8 | S | Y |
| T10 | *Escherichia coli* | KPC-3, CTX-M G1, TEM, OXA | 16 | S | Y |
| T11 | *Escherichia coli* | KPC-3 | 16 | S | Y |
| T12 | *Escherichia coli* | OXA-181, CTX-M G1, CMY, OXA | 16 | S | Y |
| T13 | *Escherichia coli* | NDM-1, CTX-M G1, CMY, TEM | 16 | S | Y |
| T14 | *Escherichia coli* | NDM-5 | 16 | S | Y |
| T15 | *Escherichia coli* | VIM-1, CTX-M G1, TEM, OXA | 16 | S | Y |
| T16 | *Escherichia coli* | CTX-M G9, DHA | 16 | S | Y |
| T17 | *Escherichia coli* | CTX-M G1, TEM, SHV | 16 | S | Y |
| T18 | *Escherichia coli* | CTX-M G1, TEM, SHV | 16 | S | Y |
| T19 | *Escherichia coli* | CTX-M G1, OXA | 16 | S | Y |
| T20 | *Escherichia coli* | CTX-M G3, TEM, SHV | 16 | S | Y |
| T21 | *Escherichia coli* | CTX-M G9, TEM | 16 | S | Y |
| T22 | *Escherichia coli* | TEM | 16 | S | Y |
| T23 | *Escherichia coli* |  | 16 | S | Y |
| T24 | *Escherichia coli* | NDM-5, TEM | 32 | R | Y |
| T25 | *Escherichia coli* | NDM-5 | 32 | R | Y |
| T26 | *Escherichia coli* | OXA-244, CTX-M G1, OXA, TEM | 32 | R | Y |
| T27 | *Escherichia coli* | OXA-244, CTX-M G9 | 32 | R | Y |
| T28 | *Escherichia coli* | OXA-244, DHA | 32 | R | Y |
| T29 | *Escherichia coli* | OXA-244, TEM | 32 | R | Y |
| T30 | *Escherichia coli* | CTX-M G1, TEM | 32 | R | Y |
| T31 | *Escherichia coli* | CTX-M G9, CMY, TEM | 32 | R | Y |
| T32 | *Escherichia coli* | TEM | 32 | S | N |
| T33 | *Escherichia coli* | OXA-244, CTX-M G1 | 64 | R | Y |
| T34 | *Escherichia coli* | OXA-484 | 64 | R | Y |
| T35 | *Escherichia coli* | CTX-M G1, CMY, TEM | 64 | R | Y |
| T36 | *Escherichia coli* | CTX-M G1, TEM | 64 | R | Y |
| T37 | *Escherichia coli* | CTX-M G1, OXA | 64 | R | Y |
| T38 | *Escherichia coli* | NDM-5, CTX-M G1, OXA | 128 | R | Y |
| T39 | *Escherichia coli* | NDM-5, CTX-M G1, OXA | 128 | R | Y |
| T40 | *Escherichia coli* | NDM-19 | 128 | R | Y |
| T41 | *Escherichia coli* | CTX-M G1, CMY, OXA, TEM | 128 | R | Y |
| T42 | *Escherichia coli* | CTX-M G1, OXA | 128 | R | Y |
| T43 | *Escherichia coli* | NDM-5, CTX-M G1, OXA | 256 | R | Y |
| T44 | *Escherichia coli* | NDM-5, CMY | 256 | R | Y |
| T45 | *Escherichia coli* | KPC-3, TEM, SHV | 256 | R | Y |
| T46 | *Escherichia coli* | OXA-48, CTX-M G9, TEM | 256 | R | Y |
| T47 | *Escherichia coli* | OXA-48, CTX-M G1, TEM, OXA | 256 | R | Y |
| T48 | *Escherichia coli* | OXA-181, CTX-M G2, TEM, OXA | 256 | R | Y |
| T49 | *Escherichia coli* | OXA-181, CTX-M G1, TEM | 256 | R | Y |
| T50 | *Escherichia coli* | OXA-181, DHA | 256 | R | Y |
| T51 | *Escherichia coli* | KPC-3, NDM-5, CMY, CTX-M G1, OXA, TEM, SHV | >256 | R | Y |
| T52 | *Escherichia coli* | NDM-5, OXA-244 | >256 | R | Y |
| T53 | *Escherichia coli* | NDM-5, CTX-M G1, OXA | >256 | R | Y |
| T54 | *Escherichia coli* | NDM-5, CMY | >256 | R | Y |
| T55 | *Escherichia coli* | OXA-48, CTX-M G9 | >256 | R | Y |
| T56 | *Klebsiella pneumoniae* | CTX-M G1, SHV | 2 | S | Y |
| T57 | *Klebsiella pneumoniae* | DHA, SHV | 2 | S | Y |
| T58 | *Klebsiella pneumoniae* | CTX-M G9, TEM, SHV | 2 | S | Y |
| T59 | *Klebsiella pneumoniae* | NDM-1, DHA, OXA, SHV | 4 | S | Y |
| T60 | *Klebsiella pneumoniae* | SHV | 4 | S | Y |
| T61 | *Klebsiella pneumoniae* | CTX-M G9, TEM, SHV | 8 | S | Y |
| T62 | *Klebsiella pneumoniae* | CTX-M G1, OXA, TEM, SHV | 16 | S | Y |
| T63 | *Klebsiella pneumoniae* | CTX-M G1, TEM, SHV | 16 | S | Y |
| T64 | *Klebsiella pneumoniae* | CTX-M G9, DHA, SHV | 16 | S | Y |
| T65 | *Klebsiella pneumoniae* | TEM, SHV | 16 | S | Y |
| T66 | *Klebsiella pneumoniae* | SHV | 16 | S | Y |
| T67 | *Klebsiella pneumoniae* | KPC-2, VEB-25, OXA, SHV | 32 | R | Y |
| T68 | *Klebsiella pneumoniae* | KPC-41, TEM, SHV | 32 | R | Y |
| T69 | *Klebsiella pneumoniae* | KPC-3, SHV | 64 | R | Y |
| T70 | *Klebsiella pneumoniae* | KPC-46, SHV | 64 | R | Y |
| T71 | *Klebsiella pneumoniae* | OXA-48, CTX-M G9, SHV | 64 | R | Y |
| T72 | *Klebsiella pneumoniae* | CTX-M G1, OXA, SHV | 64 | R | Y |
| T73 | *Klebsiella pneumoniae* | CTX-M G1, TEM, SHV | 64 | R | Y |
| T74 | *Klebsiella pneumoniae* | KPC-3, NDM-1, SHV | 128 | R | Y |
| T75 | *Klebsiella pneumoniae* | KPC-3, NDM-1, SHV | 128 | R | Y |
| T76 | *Klebsiella pneumoniae* | KPC-50, SHV | 128 | R | Y |
| T77 | *Klebsiella pneumoniae* | NDM-1, CTX-M G1, SHV | 128 | R | Y |
| T78 | *Klebsiella pneumoniae* | NDM-2, CTX-M G1, TEM, SHV | 128 | R | Y |
| T79 | *Klebsiella pneumoniae* | VIM-1, CTX-M G2, SHV | 128 | R | Y |
| T80 | *Klebsiella pneumoniae* | CTX-M G1, TEM, SHV | 128 | R | Y |
| T81 | *Klebsiella pneumoniae* | OXA-48, SHV | 256 | R | Y |
| T82 | *Klebsiella pneumoniae* | CTX-M G1, TEM, SHV | 256 | R | Y |
| T83 | *Klebsiella pneumoniae* | KPC-3, SHV | >256 | R | Y |
| T84 | *Klebsiella pneumoniae* | IMP-1, OXA, TEM, SHV | >256 | R | Y |
| T85 | *Klebsiella pneumoniae* | OXA-48, CTX-M G1, CTX-M G9, OXA, SHV | >256 | R | Y |
| T86 | *Klebsiella pneumoniae* | OXA-48, CTX-M G1, OXA, SHV | >256 | R | Y |
| T87 | *Klebsiella pneumoniae* | OXA-204, CTX-M G1, CMY, OXA, SHV | >256 | R | Y |
| T88 | *Klebsiella pneumoniae* | VIM-1, SHV | >256 | R | Y |
| T89 | *Enterobacter cloacae* |  | 16 | S | Y |
| T90 | *Enterobacter cloacae* | CTX-M G9 | 32 | R | Y |
| T91 | *Enterobacter cloacae* | NDM-1, CTX-M G1, OXA, SHV, TEM | 128 | R | Y |
| T92 | *Enterobacter cloacae* | VIM-1, OXA, TEM | 128 | R | Y |
| T93 | *Enterobacter cloacae* | VIM-1, CTX-M G9, SHV | >256 | R | Y |
| T94 | *Enterobacter cloacae* | VIM-1 | >256 | R | Y |
| T95 | *Klebsiella aerogenes* | NDM-5 | 16 | S | Y |
| T96 | *Klebsiella aerogenes* | OXA-484 | 32 | R | Y |
| T97 | *Klebsiella aerogenes* |  | 32 | R | Y |
| T98 | *Klebsiella aerogenes* | OXA-181 | 128 | R | Y |
| T99 | *Klebsiella oxytoca* |  | 4 | S | Y |
| T100 | *Klebsiella oxytoca* | CTX-M G1, TEM | 8 | S | Y |

**Table S1.** The 100 tested clinical Enterobacterale isolates, including beta-lactamase gene carriage, temocillin MICs, and rapid temocillin test results.
